# Depression, anxiety, and suicidal ideation in young adults 5 years after undergoing bariatric surgery as adolescents

**DOI:** 10.1007/s40519-020-01024-0

**Published:** 2020-10-20

**Authors:** Kajsa Järvholm, Torsten Olbers, Markku Peltonen, Claude Marcus, Carl-Erik Flodmark, Eva Gronowitz, Jovanna Dahlgren, Jan Karlsson

**Affiliations:** 1 Childhood Obesity Unit, Skåne University Hospital, Malmö, Sweden; 2 Department of Pediatrics, Institute of Clinical Sciences, Sahlgrenska Academy, University of Gothenburg, Gothenburg, Sweden; 3 Department of Clinical and Experimental Medicine, Linköping University, Linköping, Sweden; 4 National Institute for Health and Welfare, Helsinki, Finland; 5 Department of Clinical Science, Intervention and Technology, Karolinska Institutet, Stockholm, Sweden; 6 Department of Clinical Sciences in Malmö, Lund University, Lund, Sweden; 7 Region Västra Götaland, Regional Obesity Center, Sahlgrenska University Hospital, Gothenburg, Sweden; 8 University Health Care Research Center, Faculty of Medicine and Health, Örebro University, Örebro, Sweden

**Keywords:** Bariatric surgery, Adolescent, Obesity, Depression, Anxiety, Suicidal ideation

## Abstract

**Purpose:**

Metabolic and bariatric surgery (MBS) is increasingly used in adolescents. The aim was to explore symptoms of depression and anxiety in young adults over 5 years’ follow-up after undergoing MBS.

**Methods:**

Beck Depression Inventory-2 and the Beck Anxiety Inventory were used to assess symptoms of depression and anxiety in 62 patients 1, 2, and 5 years after having Roux-en-Y gastric bypass at 13–18 years of age. Mental health, eating-related problems, and weight outcomes were tested for association with suicidal ideation at the 5-year follow-up.

**Results:**

At the 5-year follow-up, the mean score for depression was 11.4 (± 12.4), indicating minimal symptoms of depression. The mean score for anxiety was 12.82 (± 11.50), indicating mild anxiety symptoms. Still, several participants reported moderate or severe symptoms of depression (26%) and anxiety (32%). Women reported more symptoms than men (*P* = 0.03 and 0.04). No significant changes were found in self-reported symptoms of depression and anxiety between the 1-year and the 5-year follow-up (*P* = 0.367 and 0.934). Suicidal ideation was reported by 16% at the 5-year follow-up. Participants reporting suicidal ideation had lost significantly less excess weight than participants without suicidal ideation (*P* = 0.009).

**Conclusion:**

Five years after adolescent MBS, a substantial minority still struggles with mental health issues, and women are more burdened than men. Our results indicate an association between less optimal weight loss and suicidal ideation 5 years after MBS. The findings emphasize the importance of offering long-term follow-up and mental health treatment several years after MBS.

**Level of evidence:**

Level III, cohort study.

**Clinical trial registration:**

The study is registered with ClinicalTrials.gov (NCT00289705). First posted February 10, 2006.

## Introduction

Obesity is associated with an increased risk of depression and suicidal ideation, especially in women [1, 2]. Western societies are characterized by an ideal of thinness [3], and children as young as 3 years associate slenderness with happiness and obesity with distress [4]. Patients seeking metabolic and bariatric surgery (MBS) may therefore hope for improved mental health following a massive weight loss [5]. MBS is a standard treatment for severe obesity in adults, and studies focusing on safety, weight loss, and the resolution of comorbidities after MBS have found similar favourable outcomes in adolescents and adults [6, 7].

Many patients, including adolescents, report reduced symptoms of depression and improved health-related quality of life (HRQoL) during the first year after MBS [8–11]. However, weight loss does not appear to improve long-term mental health in an unambiguous way [12–14]. Studies of mental health outcomes after adolescent MBS in young adulthood are limited, but previous studies from our Swedish group and US cohorts indicate that adolescents and young adults undergoing MBS are more psychologically vulnerable than middle-aged patients [14–17].

Less data is available on the treatment effects of MBS on symptoms of anxiety. The findings in middle-aged adults are mixed, and it has been suggested that anxiety is less associated with weight than depression [9]. However, when evaluating MBS as a treatment for adolescents and young adults, it is necessary to study outcomes related to anxiety, as anxiety disorders have an earlier age of onset (median 11 years) than mood disorders (median 30 years) [18]. We have previously shown substantially reduced symptoms of anxiety in adolescents 2 years after MBS [11].

Several studies have reported an increased risk of attempted and even completed suicide after MBS [13, 16, 19]. Risk factors currently identified for self-harm and suicide in adults after MBS are male sex, a history of psychiatric disorders, and sleep difficulties [20]; however, a US study of suicidal behaviour (ideation, plans, and attempts) in adolescents over 4 years after MBS identified several baseline and post-operative risk factors including female sex, lower HRQoL, more weight-related problems, and loss of control over eating [21]. Today, there is consensus about the increased risk of suicidal behaviour after MBS and the necessity of screening, but further studies are needed to better understand the possible relationship between risk factors and post-MBS suicidal behaviours [22].

The aim of the present study was to explore symptoms of depression and anxiety in young adults over 5 years’ follow-up after undergoing MBS. Based on long-term studies in adolescents and adults, we hypothesized that mental health would not improve from 1 to 5 years after MBS. A second aim was to explore factors associated with suicidal ideation 5 years after surgery in this sample.

## Materials and methods

### Participants

All participants were from the Swedish multi-centre study Adolescent Morbid Obesity Surgery (AMOS). The primary endpoints were the safety and efficacy of Roux-en-Y gastric bypass (RYGB) in adolescents aged 13–18.

Between 2006 and 2009, 81 adolescents from three childhood obesity units in Sweden were included in AMOS. All adolescents had laparoscopic RYGB from the same surgical team. In the present study, we used data from the 62 adolescents who filled in self-report questionnaires assessing symptoms of depression and anxiety at the 5-year follow-up.

AMOS has been described in more detail elsewhere [23]. Inclusion criteria were body mass index (BMI) ≥ 40 kg/m^2^ or ≥ 35 kg/m^2^ with an obesity-related comorbidity. The adolescents should have been in conservative weight-loss treatment for ≥ 1 year before entering the study. Exclusion criteria were few but included ongoing drug abuse and serious psychiatric disorders such as psychosis, severe depression, and self-induced vomiting (as this could indicate an untreated severe eating disorder).

The AMOS study was approved by the Central Ethical Review Board of Gothenburg (registration number 532-04) and is registered in Clinical Trials.gov (NCT00289705). Informed consent was obtained from all participants and their caregivers.

### Main outcome variables

#### Beck depression inventory 2

The Beck Depression Inventory-2 (BDI-2) is a 21-item questionnaire assessing symptoms of depression over the previous 2 weeks in adolescents ≥ 13 years and adults [24]. Total scores range from 0 to 63, and higher scores indicate more symptoms of depression. Scores of 0–13 indicate minimal depression, 14–19 mild depression, 20–28 moderate depression, and 29–63 severe depression [24]. One item explicitly assesses suicidal ideation. Statement 1 indicates no suicidal ideation (I do not have any thoughts of killing myself), statement 2 indicates passive suicidal ideation (I have thoughts of killing myself, but I would not carry them out), and statements 3 and 4 indicate active suicidal ideation (I would like to kill myself and I would kill myself if I had the chance). BDI-2 is widely used in different clinical settings and is a useful measure of depressive symptoms in MBS samples [25]. Its internal consistency reliability in the present study was excellent (Cronbach’s *α* = 0.953).

#### Beck anxiety inventory

The Beck Anxiety Inventory (BAI) is a 21-item questionnaire assessing anxiety symptoms over the previous week in adolescents ≥ 17 years and adults [26]. The BAI was developed to capture those anxiety symptoms that are least shared with depressive disorders to distinguish between these often associated conditions. Total scores range from 0 to 63, and higher scores indicate more anxiety symptoms. Scores of 0–7 indicate minimal anxiety, 8–15 mild anxiety, 16–25 moderate anxiety, and 26–63 severe anxiety [26]. Its internal consistency reliability in the present study was excellent (Cronbach’s *α* = 0.929).

### Variables tested for association with suicidal ideation at 5 years

#### Rosenberg Self-Esteem

Rosenberg Self-Esteem (RSE) comprises 10 items assessing global self-esteem in adolescents and adults [27]. Total scores range from 0 to 30, and higher scores represent higher self-esteem. The RSE is one of the questionnaires most used to assess general self-esteem, and its internal consistency reliability was excellent in the present sample (Cronbach’s *α* = 0.931).

#### Mood Adjective Check List

The Mood Adjective Check List (MACL) comprises 38 adjectives describing positive and negative mood states [28]. Three basic dimensions of mood are assessed: calmness versus tension, activation versus deactivation, and pleasantness versus unpleasantness. An overall mood score is also calculated. Scores range from 1 to 4, and a higher score corresponds to a more positive mood. The MACL has previously been used to assess mood in MBS patients [9], and its internal consistency reliability for overall mood was excellent in the present sample (Cronbach’s *α* = 0.959).

#### The Obesity-related Problems scale

The Obesity-related Problems (OP) scale consists of 14 items assessing psychosocial problems related to weight and body shape. Total scores range from 0 to 100, and a higher score indicates more problems [29]. The OP is a valid measure of problems related to weight and shape in people with obesity and has been used to assess outcomes after MBS [29]. Its internal consistency reliability was excellent in the present sample (Cronbach’s *α* = 0.954).

#### Binge Eating Scale

The Binge Eating Scale (BES) is a 16-item questionnaire assessing behaviours related to binge eating [30]. Total scores range from 0 to 46, and higher scores correspond to more binge eating. The BES was developed to assess binge eating in subjects with obesity [30], and it is a valid screening instrument for binge eating in MBS samples [31]. Its internal consistency reliability was good in the present sample (Cronbach’s *α* = 0.887).

#### Three-Factor Eating Questionnaire R-21

The Three-Factor Eating Questionnaire-R21 (TFEQ) consists of 21 items and assesses three potentially problematic eating behaviours: emotional eating, uncontrolled eating, and restrained eating [32]. Scores range from 0 to 100, and higher scores indicate more of the assessed eating behaviour. The TFEQ has previously been used to assess eating behaviour in MBS samples and has good psychometric properties [32]. In the present sample, its internal consistency reliability for emotional eating was excellent (Cronbach’s *α* = 0.950), for uncontrolled eating, good (Cronbach’s *α* = 0.872), and for cognitive restraint, questionable (Cronbach’s *α* = 0.674).

#### Short Form-36 version 2

The Short Form-36 version 2 (SF-36v2) is a generic measure that assesses aspects of physical and mental HRQoL and generates two summary measures: the physical and mental component summary scores (PCS and MCS) [33]. The summary scores are norm-based, with a mean of 50. A higher score represents a better HRQoL. The SF-36v2 is one of the most widely used instruments for assessing HRQoL in clinical studies, and its reliability estimates from the Swedish population sample are 0.92 for PCS and 0.88 for MCS [34].

### Procedure

The participants were assessed at baseline, and at 1, 2, and 5 years after gastric bypass. The RSE, MACL, OP, BES, TFEQ, and SF-36 were administrated by study coordinators, while the BDI-2 and BAI were administrated by clinical psychologists. If no psychologist was available on the day of assessment, the BDI-2 and BAI were not collected. Therefore, data are missing for these questionnaires for 19 participants from the 5-year follow-up.

At baseline, the Beck Youth Inventories (BYI) developed for children and adolescents aged 9–18 years, were used to assess symptoms of depression and anxiety. BDI-2 and BAI were then used to assess symptoms of depression and anxiety at follow-ups 1, 2, and 5 years after MBS. BAI was administrated to all participants including those who had not turned 17 years at the day of assessment, (1-year follow-up: *n* = 7; range 15.5–16.9 years and 2-year follow-up *n* = 1; 16.5 years).

### Statistical analysis

Descriptive data are presented as mean and standard deviation. Gender differences were analysed with Fisher’s exact test (dichotomous variables) or independent sample *t*-test (continuous variables). Changes over time were analysed using multilevel mixed-effects regression models. Observations were considered nested within the individual. Therefore, standard errors and confidence intervals were calculated controlling for the repeated measurements. Independent samples *t*-test was also used to compare differences between participants with continued or deteriorating mental health and with and without suicidal ideation 5 years after surgery. Statistical analyses were carried out using the Stata statistical package 15.1 (StataCorp. 2017, Stata Statistical Software: Release 15, College Station, TX: StataCorp LLC) and SPSS 25 (IBM Corp. Released 2017 IBM SPSS Statistics for Windows, Version 25.0. Armonk, NY: IBM Corp).

## Results

### Sample characteristics

Table 1 presents sample characteristics.

**Table 1:** Sample characteristics

	*n* = 62	
Sex, female; *n* (%)	41 (66)	
Age at baseline, years; mean (± SD)	16.9 (± 1.22)	
BMI at baseline, kg/m^2^; mean (± SD)	45.9 (± 6.39)	
BMI at 5-year follow-up, kg/m^2^; mean (± SD)	32.5 (± 6.03)	
		Norm or cut-off for reference
Rosenberg Self-Esteem at baseline; mean (± SD)	19.1 (± 7.3)	Cut-off: < 15 low self-esteem
MACL overall mood at baseline; mean (± SD)	2.71 (± 0.43)	3.17 (± 0.43) norm group aged 18–25
Obesity-related Problems at baseline; mean (± SD)	49.2 (± 23.3)	Cut-off: < 40 mild, 40–59 moderate, ≥ 60 severe impairment
Binge Eating Scale at baseline; mean (± SD)	15.4 (± 7.0)	Cut-off: > 17 binge eating
TFEQ UE at baseline; mean (± SD)	46.7 (± 19.0)	33.2 (± 17.6) norm group aged 16–17^a^
TFEQ CR at baseline; mean (± SD)	40.8 (± 18.8)	24.1 (± 20.7) norm group aged 16–17^a^
TFEQ EE at baseline; mean (± SD)	40.8 (± 25.1)	18.0 (± 21.9) norm group aged 16–17^a^
MCS at baseline; mean (± SD)	44.2 (± 12.1)	49,4 (± 9,1) norm group aged 15–19
PCS at baseline; mean (± SD)	42.9 (± 9.6)	53,5 (± 6,7) norm group aged 15–19

*SD* standard deviation, *BMI* body mass index, *MACL* mood adjective check list, *TFEQ-R21* three-factor eating questionnaire, *UE* uncontrolled eating, *CR* cognitive restraint, *EE* emotional eating, *MCS* mental component summary from SF-36, *PCS* physical component summary from SF-36

^a^Unpublished reference for Swedish adolescents

### Comparison between adolescents with and without BDI-2 and BAI data at 5 years

There were no significant differences at baseline or at the 5-year follow-up in BMI, age, sex, or any other variable included in the present study (*P* > 0.05; Table 2) between the adolescents (*n* = 62) who completed the BDI-2 and BAI at 5 years and those who did not (*n* = 19).

**Table 2:** Comparison between participants in the AMOS-study with and without assessment of depression and anxiety at the 5-year follow-up

Variable	With BDI2 and BAI data at 5 year, *n* = 62 Percent or mean (± SD)	Without BDI2 and BAI data at 5 year, *n* = 19 Percent or mean (± SD)	*P* value
Sex	66% girls	63% girls	0.79
BMI at baseline	45.9 (± 6.4)	44.0 (± 4.6)	0.227
BMI at 5 years	32.5 (± 6.0)	31.7 (± 7.1)	0.625
Age at baseline	16.9 (± 1.2)	16.7 (± 1.2)	0.658
RSE at baseline	19.1 (± 7.3)	18.4 (± 8.2)	0.733
RSE at 5 years	21.6 (± 7.1)	21.1 (± 8.7)	0.839
MACL OM at baseline	2.71 (± 0.43)	2.65 (± 0.47)	0.623
MACL OM at 5 years	2.84 (± 0.56)	2.72 (± 0.69)	0.517
OP at baseline	49.2 (± 23.3)	53.3 (± 27.5)	0.572
OP at 5 years	37.5 (± 27.0)	34.4 (± 35.2)	0.707
BES at baseline	15.4 (± 7.0)	13.8 (± 10.9)	0.471
BES at 5 years	9.7 (± 8.4)	7.7 (± 7.9)	0.419
TFEQ UE at baseline	46.7 (± 19.0)	38.9 (± 25.2)	0.162
TFEQ UE at 5 years	29.1 (± 21.0)	22.5 (± 18.5)	0.282
TFEQ CR at baseline	40.8 (± 18.8)	36.1 (± 18.8)	0.360
TFEQ CR at 5 years	52.2 (± 21.3)	41.4 (± 31.9)	0.125
TFEQ EE at baseline	40.8 (± 25.1)	35.5 (± 30.2)	0.459
TFEQ EE at 5 years	26.7 (± 28.1)	18.3 (± 22.6)	0.295
MCS at baseline	44.2 (± 12.1)	43.7 (± 12.3)	0.905
MCS at 5 years	45.5 (± 11.6)	43.7 (± 14.4)	0.629
PCS at baseline	42.9 (± 9.6)	45.8 (± 9.9)	0.281
PCS at 5 years	48.6 (± 10.1)	49.1 (± 11.0)	0.851

Comparison with Fischer’s exact test (sex) or Independent sample *t*-test

*BDI-2* Beck Depression Inventory 2, *BAI* Beck Anxiety Inventory, *SD* standard deviation, *BMI* body mass index, *RSE* Rosenberg Self-Esteem scale, *MACL* mood adjective check list, *OM* overall mood, *OP* obesity-related problems, *BES* Binge Eating Scale, *TFEQ* three-factor eating questionnaire, *UE* uncontrolled eating, *CR* cognitive restraint, *EE* emotional eating, *MCS* mental component summary, *PCS* physical component summary

### Self-reported symptoms of depression and anxiety

Five years after surgery the mean score for symptoms of depression (BDI-2) was in the minimal range (11.4 ± 12.4). Women reported significantly more symptoms than men (13.8 ± 13.6 vs. 6.7 ± 7.8; *P* = 0.03). The mean score for symptoms of anxiety (BAI) was in the mild range (12.82 ± 11.50), and women reported significantly more symptoms than men (14.9 ± 11.7 vs. 8.7 ± 10.0; *P* = 0.04). Number of subjects in each clinical category at 5 years are presented in Table 3. After 5 years, 26% and 32% reported moderate to severe symptoms of depression and anxiety, respectively.

**Table 3:** Number and percent of adolescents in each clinical category 5 years after gastric bypass

	Minimal symptoms *n* (%)	Mild symptoms *n* (%)	Moderate symptoms *n* (%)	Severe symptoms *n* (%)
Depressive symptoms—all (*n* = 62)	42 (67.7%)	4 (6.4%)	8 (12.9%)	8 (12.9%)
Depressive symptoms—women (*n* = 41)	24 (58.5%)	3 (7.3%)	7 (17.1%)	7 (17.1%)
Depressive symptoms—men (*n* = 21)	18 (85.7%)	1 (4.8%)	1 (4.8%)	1 (4.8%)
Anxiety symptoms—all (*n* = 62)	24 (38.7%)	18 (29.0%)	12 (19.4%)	8 (12.9%)
Anxiety symptoms—women (*n* = 41)	11 (26.8%)	15 (36.6%)	9 (22.0%)	6 (28.6%)
Anxiety symptoms—men (*n* = 21)	13 (61.9%)	3 (14.3%)	3 (14.3%)	2 (9.5%)

**Fig. 1:**
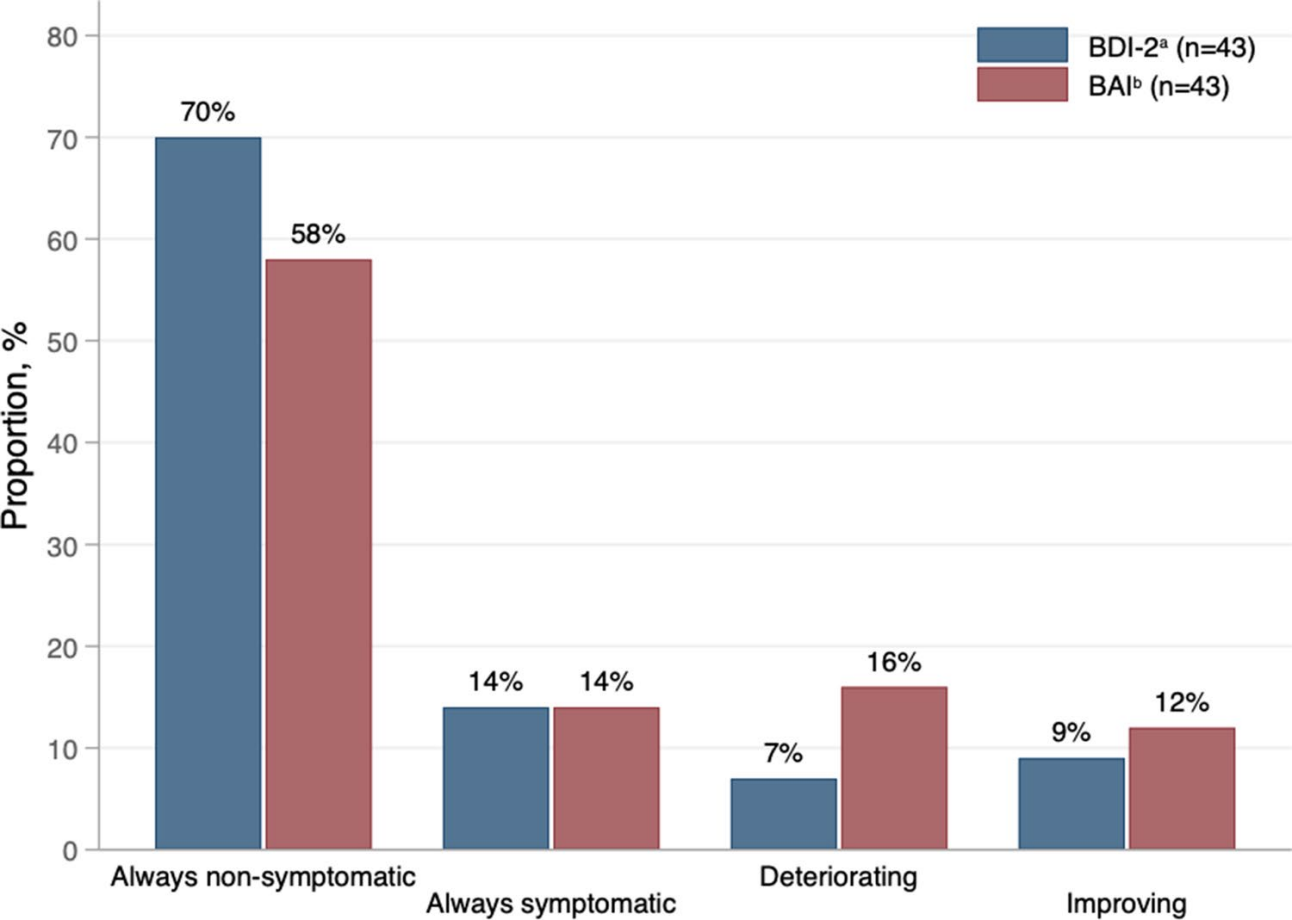
Individual change or stability in symptoms of depression and anxiety between 2 and 5 years after gastric bypass. *BDI-2* Beck Depression Inventory 2, *BAI* Beck Anxiety Inventory. **a** Always non-symptomatic: BDI-2 < 20 (i.e., minimal or mild) at both follow-ups (*n* = 30); always symptomatic: BDI-2 ≥ 20 (i.e., moderate to severe) at both follow-ups (*n* = 6); deteriorating: BDI-2 < 20 at 2-year follow-up and ≥ 20 at 5-year follow-up (*n* = 3); improving: BDI-2 ≥ 20 at 2-year follow-up and < 20 at 5-year follow-up (*n* = 4). **b** Always non-symptomatic: BAI < 16 (i.e., minimal or mild) at both follow-ups (*n* = 25); always symptomatic: BAI ≥ 16 (i.e., moderate to severe) at both follow-ups (*n* = 6); deteriorating: BAI < 16 at 2-year follow-up and ≥ 16 at 5-year follow-up (*n* = 7); improving: BAI ≥ 16 at 2-year follow-up and < 16 at 5-year follow-up (*n* = 5)

There were no significant changes in self-reported symptoms of depression and anxiety between the 1, 2, and 5 years’ follow-up (Table 4). Numbers of participants with stable or changing clinical categories from the 2- to the 5-year follow-up are presented in Fig. 1 for the 43 participants with data from both follow-ups, showing that a majority (84% for depressive symptoms and 72% for anxiety symptoms) of participants remained in the same clinical category from the 2- to the 5-year follow-up. According to BDI-2, 9 participants (21%) were either in the always symptomatic or the deteriorated group, and according to BAI 13 participants (30%) were either in the always symptomatic or the deteriorated group. In total, 14 participants (33%) were in the always symptomatic or the deteriorated group according to BDI-2 and/or BAI. There was no significant difference in sex distribution between participants in the always symptomatic/deteriorated groups and participants who were never symptomatic or improved (*P* = 0.49). However, the always symptomatic/deteriorated groups had less optimal weight outcomes at 5 years (% excess BMI loss 52.5 vs. 77.2, *P* = 0.001).

**Table 4:** Self-reported symptoms of depression and anxiety at follow-ups

	1 year *n* = 33 mean (95% CI)	2 year *n* = 43 mean (95% CI)	5 year *n* = 62 mean (95% CI)	5 year vs. 1 year *P* value	5 year vs. 2 year *P* value
BDI-2	9.0 (5.7–12.2)	11.5 (8.5–14.5)	10.6 (7.6–13.6)	0.367	0.610
BAI	12.5 (9.4–15.5)	12.6 (9.8–15.4)	12.6 (9.9–15.3)	0.934	0.998

Data presented as mixed-model mean and 95% confidence interval

*BDI-2* Beck Depression Inventory 2, *BAI* Beck Anxiety Inventory

### Suicidal ideation after 5 years

At the 5-year follow-up, 10 (16%) participants reported suicidal ideation during the previous 14 days; of those, six (9%) reported passive, and four (6%) active, suicidal ideation. Nine were women (22% of the women reported suicidal ideation vs. 5% of the men), but the difference in the sex distribution did not reach statistical significance (*P* = 0.14).

Variables related to mental health, eating problems, and HRQoL at baseline and 1- and 2-year follow-ups were compared among participants with and without suicidal ideation at year 5 (Table 5). The mood dimension calmness/tension and the SF-36 physical health component summary score differed significantly between the groups at baseline, but no other variables, including those assessing eating-related problems, were significantly different. Most of the general mental health variables analysed at the 1-year follow-up, and all general mental health variables analysed at the 2-year follow-up, showed significantly worse general mental health in the participants who had suicidal ideation at the 5-year follow-up. However, among the eating-related variables, only cognitive restraint at 1 year and emotional eating at 2 years differed significantly between the groups (Table 5). Weight outcome was also compared between the groups at year 5 (Table 5), demonstrating poorer weight loss among participants reporting suicidal ideation.

**Table 5:** Mental health, eating-related problems, health-related quality of life, and weight outcome analyzed separately for participants with and without suicidal ideation 5 years after surgery

Variable	No suicidal ideation at 5 years	*n*	Suicidal ideation at 5 years	*n*	*P* value	Norm or cut-off for reference
Baseline						
BMI	45.42 (6.45)	52	48.42 (5.71)	10	0.176	
RSE	19.04 (6.76)	50	19.30 (10.20)	10	0.920	Cut-off: < 15 low self-esteem
MACL p	2.94 (0.49)	50	2.62 (0.70)	10	0.095	
MACL a	2.64 (0.43)	50	2.55 (0.62)	10	0.580	
MACL c	2.69 (0.51)	50	2.30 (0.64)	10	0.035*	
MACL o	2.76 (0.38)	50	2.49 (0.60)	10	0.076	3.17 (± 0.43) norm group aged 18–25
OP	48.52 (25.08)	50	52.86 (33.14)	10	0.639	Cut-off: < 40 mild, 40–59 moderate, ≥ 60 severe impairment
BES	15.38 (7.01)	50	15.44 (6.62)	10	0.980	Cut-off: > 17 binge eating
TFEQ UE	47.77 (18.18)	49	41.48 (22.75)	10	0.344	33.2 (± 17.6) norm group aged 16–17^a^
TFEQ CR	41.27 (17.53)	49	38.33 (25.18)	10	0.657	24.1 (± 20.7) norm group aged 16–17^a^
TFEQ EE	38.44 (22.70)	49	52.22 (33.56)	10	0.114	18.0 (± 21.9) norm group aged 16–17^a^
MCS	45.44 (11.32)	49	37.81 (14.48)	10	0.069	49,4 (± 9.1) norm group aged 15–19
PCS	44.23 (8.71)	49	36.35 (11.68)	10	0.017*	53.5 (± 6.7) norm group aged 15–19
1 year						
BMI	30.53 (3.96)	52	33.22 (5.06)	10	0.065	
RSE	22.84 (6.57)	51	18.90 (7.06)	10	0.092	Cut-off: < 15 low self-esteem
MACL p	3.10 (0.52)	50	2.71 (0.65)	10	0.040*	
MACL a	2.84 (0.50)	50	2.69 (0.71)	10	0.421	
MACL c	2.86 (0.46)	50	2.42 (0.69)	10	0.013*	
MACL o	2.93 (0.43)	50	2.61 (0.66)	10	0.050*	3.17 ( 0.43) norm group aged 18–25
OP	25.81 (20.12)	50	50.95 (34.53)	10	0.002*	Cut-off: < 40 mild, 40–59 moderate, ≥ 60 severe impairment
BES	6.69 (5.56)	51	10.70 (10.83)	10	0.086	Cut-off: > 17 binge eating
TFEQ UE	25.05 (17.32)	51	21.85 (18.76)	10	0.600	33.2 (± 17.6) norm group aged 16 to 17^a^
TFEQ CR	47.82 (19.57)	51	61.11 (12.28)	10	0.044*	24.1 (± 20.7) norm group aged 16–17^a^
TFEQ EE	21.35 (20.59)	51	27.22 (23.78)	10	0.424	18.0 (± 21.9) norm group aged 16–17^a^
MCS	47.53 (8.50)	51	38.26 (16.14)	10	0.010*	49.4 (± 9.1) norm group aged 15–19
PCS	52.33 (5.91)	51	47.99 (10.24)	10	0.068	53.5 (± 6.7) norm group aged 15–19
2 year follow-up						
BMI	29.65 (4.11)	52	32.72 (4.10)	10	0.034*	
RSE	23.17 (5.87)	48	15.63 (9.13)	8	0.003*	Cut-off: < 15 low self-esteem
MACL p	3.10 (0.49)	48	2.36 (0.80)	7	0.001*	
MACL a	2.76 (0.58)	48	2.27 (0.70)	8	0.036*	
MACL c	2.85 (0.56)	48	2.11 (0.62)	8	0.001*	
MACL o	2.90 (0.48)	48	2.22 (0.66)	8	0.001*	3.17 (± 0.43) norm group aged 18–25
OP	28.52 (18.49)	48	59.52 (28.63)	8	< 0.001*	Cut-off: < 40 mild, 40–59 moderate, ≥ 60 severe impairment
BES	7.87 (5.86)	48	10.88 (9.76)	8	0.233	Cut-off: > 17 binge eating
TFEQ UE	28.01 (17.67)	48	37.04 (17.93)	8	0.187	33.2 (± 17.6) norm group aged 16–17^a^
TFEQ CR	43.87 (19.99)	48	56.25 (11.28)	8	0.095	24.1 (± 20.7) norm group aged 16–17^a^
TFEQ EE	23.15 (20.12)	48	55.56 (31.15)	8	< 0.001*	18.0 (± 21.9) norm group aged 16–17^a^
MCS	45.41 (11.61)	51	28.02 (17.58)	8	0.001*	49.4 (± 9.1) norm group aged 15–19
PCS	51.26 (8.15)	51	49.93 (11.84)	8	0.688	53.5 (± 6.7) norm group aged 15–19
5 year follow-up						
% weight loss	29.07 (12.16)	52	20.26 (11.77)	10	0.039*	< 20% sub-optimal weight loss
% excess BMI loss	68.58 (26.29)	52	44.12 (25.21)	10	0.009*	

No correction for multiple testing was made as the analyzes were exploratory

*RSE* Rosenberg Self-Esteem (range 0–30), *MACL* mood adjective checklist (range 1–4), *p*: pleasantness; *c* calmness, *a* activation, *o* overall mood, *OP* the obesity-related problems scale (range 0–100), *BES* Binge Eating Scale, *TFEQ* three-factor eating questionnaire R21(range 0–100), *UE* uncontrolled eating, *CR* cognitive restraint, *EE* emotional eating, *MCS* mental component summary (range 0–100), *PCS* physical component summary (range 0–100), *BMI* body mass index

^a^Unpublished reference for Swedish adolescents

*Significant *P* value

## Discussion

This study adds to the limited knowledge about mental health in young adults who have undergone MBS as adolescents by presenting the frequency of symptoms of depression and anxiety assessed at 1, 2, and 5 years after MBS.

The proportion of young adults reporting current elevated symptoms of depression (26%) and anxiety (32%) at the 5-year follow-up after MBS was higher than expected from the estimated 12-month general European prevalences of 6.9% for depression and 14% for anxiety [35]. When adolescents in the present study were assessed at baseline using the adolescent questionnaire BYI, 24% reported highly elevated symptoms of depression and 19% reported highly elevated symptoms of anxiety [11]. Even if a direct comparison is not possible due to the differences in assessment, the findings in the present study support the main conclusion of our previous study using register data: adolescent MBS should not be expected to alleviate mental health problems [14].

The frequency of symptoms of depression and anxiety in the present study of adolescents was also higher than in operated adults, who also report more such symptoms after MBS than a reference population [9]. Four years after MBS, approximately 12% and 15% of adults, respectively, reported clinically elevated symptoms of depression and anxiety [12]. In the Swedish Obese Subjects study, 15% and 24% reported scores indicating probable clinical depression and anxiety disorder 10 years after MBS [9].

Adolescents and young adults with severe obesity, whether or not they undergo MBS, are a psychologically vulnerable group [36, 37]. Some US studies indicate equal or better mental health in adolescents undergoing MBS compared with age-matched peers with severe obesity undergoing lifestyle treatment [21, 38]. Other US studies and our previous findings from Sweden, however, show equal or worse mental health in the MBS group compared with a control group of conventionally treated adolescents with severe obesity [14, 39]. Therefore, regardless of the type of treatment, it is important to address mental health issues when treating adolescents and young adults for severe obesity.

The vulnerability to mental health problems of adolescents with former or current severe obesity is poorly understood. Experiences of bullying and stigmatization may be a part of the explanation, along with the high prevalence of attention deficit hyperactivity disorder in this group [40, 41]. In the present study, women reported more symptoms of depression and anxiety than men, a sex difference that is also found in community samples [35].

Previous studies of adults have reported stabilization or decline in mental health after initial improvement after MBS [9, 10]. In line with our hypothesis, we found relatively stable levels of self-reported symptoms of depression and anxiety from the 1- to the 5-year follow-up, which is also supported by results from individual trajectories in the 2- to the 5-year follow-up in 43 adolescents. Most participants remained in the same clinical category as at the previous assessment. However, patients who always report elevated symptoms or deterioration of their mental health warrant clinical attention, and in the present study, this group constituted a third of the sample. Beyond offering mental health treatment, this group might also need interventions to optimize their weight loss. However, at the 2-year follow-up in AMOS, we were not able to find any differences in weight loss between adolescents with poor mental health and those with average or good mental health [15]. This finding highlights the necessity of prolonged follow-up to improve both physical and mental health outcomes.

The frequency of current suicidal ideation (16%) at the 5-year follow-up was higher than the reported general 12-month prevalence of 8.3% in young adults [42]. The proportion with suicidal ideation was also higher than in a US study including both surgically and non-surgically treated adolescents with severe obesity followed up over 4 years [21]. Four years after treatment initiation, 3.7% in the surgical group and 11.5% in the non-surgical group reported any current suicidal ideation according to BDI-2. The frequency was also higher than in previous findings in adults after MBS, where 6.6% reported self-harm/suicidal ideation at the 5-year follow-up [43]; however, the frequency is about the same as that reported by participants in the same sample at the 2-year follow-up [44].

In our small sample, we were able to detect only two variables at baseline that differed significantly between patients reporting and not reporting suicidal ideation. The participants with suicidal ideation 5 years after MBS experienced more tension and worse physical health at baseline. In young population samples, there is an association between binge eating disorder and suicidality; however, the association is best explained by comorbid psychopathology. Notably, binge eating disorder in adolescents mostly proceeds suicidal behaviours, whereas the opposite is more common in adults [45]. In line with the temporal relationship suggested for adolescents, two previous studies on adolescent MBS, including the 2-year follow-up in AMOS, reported that baseline binge eating and loss of control over eating were related to suicidal behaviour in adolescents at 2 and 4 years after MBS, respectively [21, 44]. The present study, however, could not confirm the association between binge eating before surgery and suicidal ideation after, as baseline binge eating was similar in patients with and without suicidal ideation 5 years after surgery (15.44 vs. 15.38, *P* = 0.980).

There was a significant association between sub-optimal weight loss and suicidal ideation at the 5-year follow-up in our sample, but no information regarding possible causality. This contrasts with findings from adult samples, where patients with suicide or non-fatal self-harming behaviours had similar or greater weight loss than those with no such behaviours [19]. Also, in the US report on adolescents after MBS, suicidal behaviour was not predicted by weight loss [21]. Still, previous research has shown an increased risk of suicidal ideation, but not attempted suicide, in adolescents with obesity, and the risk is more increased with severe obesity [46]. It is therefore relevant to continue to study the impact of less weight loss on suicidal thoughts in adolescents and young adults.

The high frequency of mental health problems reported by the participants in the present study might have several explanations. Adolescents and young adults are more vulnerable than adults to mental health problems [18], which may partly explain the differences. Middle-aged and adolescent MBS samples may also differ systemically in other ways. A majority of adolescents seeking MBS have had severe obesity throughout childhood, which might not characterize all adult MBS patients, and many adolescents seeking MBS have experienced stigmatization and bullying from an early age, severely impacting their mental health [4, 40].

The discrepancy between our findings and those of some of the US studies [21, 38] might be explained by differences in patient selection. The exclusion criteria in the present study were few, and a majority of the adolescents who opted in were accepted for surgery. Swedish health care is also publicly funded, reducing the chance of selection bias due to socioeconomic resources. Thus, our findings illustrate that, with a broadly selected sample, a substantial minority of young adults struggle with obvious mental health problems after adolescent MBS. These findings have several clinical implications; for example, adolescents seeking MBS must be informed that any mental health problems they have will probably persist even after major weight loss, and the health care system must be able to screen for patients in need of additional mental health treatment.

Limitations of the study include missing data from a substantial proportion of the AMOS participants and the collection of data solely through questionnaires. We used a single item to assess suicidal ideation and assessed the main outcome variables only at the follow-ups. The sample size was small, and the findings must be considered preliminary and interpreted with caution. More studies with larger samples are necessary to reach any firm conclusions about the prevalence of symptoms of depression and anxiety, as well as suicidal ideation, in young adults after MBS. Because only ten participants reported suicidal ideation, all associations should be considered exploratory and evaluated cautiously; the non-significant associations in particular should not be considered evidence of a lack of association.

## Conclusion

Many adolescents with severe obesity might expect their mental health to improve after MBS, but a substantial proportion keep struggling with mental health problems 5 years after their operation. As in general population samples, young women were more affected by mental health problems than young men. Mental health problems, including suicidal ideation, appear to be overrepresented and more frequent in adolescents 5 years after undergoing MBS than in adults. Thus, assessment of mental health after MBS is especially important in younger patients, and psychological follow-ups should also be offered after their transition from paediatric to adult care.

## What is already known on this subject

Adolescents seeking bariatric surgery frequently report mental health problems. Mental health improves during the first year after surgery, but long-term follow-up in this group is scarce.

## What this study adds

Five years after bariatric surgery, a substantial minority struggles with mental health issues, including suicidal ideation. Psychological follow-ups are especially important in younger patients.
